# Tracheobronchopathia osteochondroplastica: A rare case report

**DOI:** 10.1097/MD.0000000000047049

**Published:** 2026-01-23

**Authors:** Chuchu Xu, Xiaoqiong Wang, Xiaona Yin, Xi Wang, Xiaoyue Liu, Lijuan Zhou, Fangbin Du, Yinling Jiang, Dongmei Su, Yongsheng Wang

**Affiliations:** a Department of Respiratory and Critical Care Medicine, The Second People’s Hospital of Hefei, Hefei Hospital Affiliated to Anhui Medical University, Hefei, Anhui Province, China; b Department of Respiratory and Critical Care Medicine, Bengbu Medical University, Bengbu, Anhui Province, China.

**Keywords:** airway stenosis, benign airway disease, tracheal calcification, Tracheobronchopathia osteochondroplastica disease, tracheoscopy

## Abstract

**Rationale:**

Tracheobronchopathia osteochondroplastica is a rare and complex disease usually associated with airway narrowing and dyspnea. Due to its complexity and rarity, its diagnosis and treatment have been poorly reported in clinical practice; therefore, we report a typical case to enhance the understanding and awareness of this disease.

**Patient concerns:**

The patient in this case is a 55-year-old male complaining of progressive dyspnea and cough with infection as a trigger. These symptoms have been progressively worsening over the last few months. In conjunction with imaging and tNGS, bronchoscopy was performed to rule out other respiratory conditions.

**Diagnoses:**

Tracheobronchopathia osteochondroplastica.

**Interventions:**

Antibiotic anti-infective treatment was given along with hormonal anti-inflammatory, bronchodilator, salmeterol ticarcoson inhaler inhalation application, bronchoscopic cryotherapy and laser therapy.

**Outcomes:**

The patient’s symptoms of chest tightness, cough and phlegm were relieved after treatment. Initial recovery following discharge was satisfactory, with no recurrence of dyspnea or coughing symptoms.

**Lessons:**

The aim of this study was to increase the awareness of clinical respiratory physicians about ossifying osteochondromatosis of the trachea.

## 1. Introduction

Tracheobronchopathia osteochondroplastica (TO) is a rare, idiopathic, nonneoplastic disease in which multiple submucosal osseous or cartilaginous nodules are seen on the anterior and lateral walls of the trachea and bronchi.^[1]^ It is a rare benign disease of the airways with a prevalence ranging from 0.01% to 0.80%,^[2]^ while recent studies have shown a prevalence of about 0.09%.^[3]^ The clinical manifestations and symptoms of this disease are not specific, which can easily lead to misdiagnosis or missed diagnosis clinically. Bronchoscopy is the standard method to confirm the diagnosis of TO, and there is no specific treatment for TO. Although rare, reporting this condition is crucial for the following reasons: early diagnosis presents uncertainties, data on optimal treatment strategies is limited, and undetected cases may lead to severe complications. This paper reports a case of TO and its diagnostic and therapeutic experience.

## 2. Case presentation

**Figure 1.**
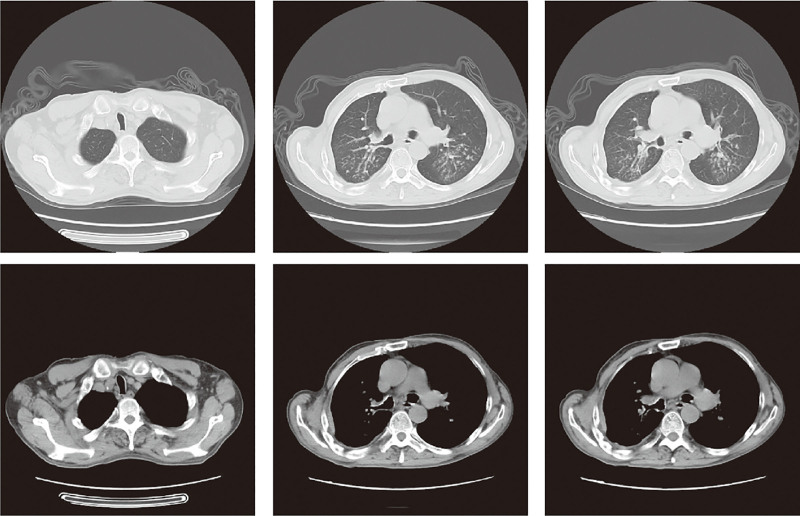
Chest CT showing both lungs show blurred texture enhancement. Multiple patchy opacities distributed along bronchial courses are visible in both lungs. Multiple bronchial walls are thickened with calcification. Partial atelectasis is noted in the middle and lower lobes of the right lung and the lower lobe of the left lung. No significantly enlarged mediastinal lymph nodes are visible. The thoracic cage is symmetrical. No definite pleural effusion is visible within the thoracic cavity. Diffuse thickening of the right pleura is present. CT = computed tomography.

**Figure 2.**
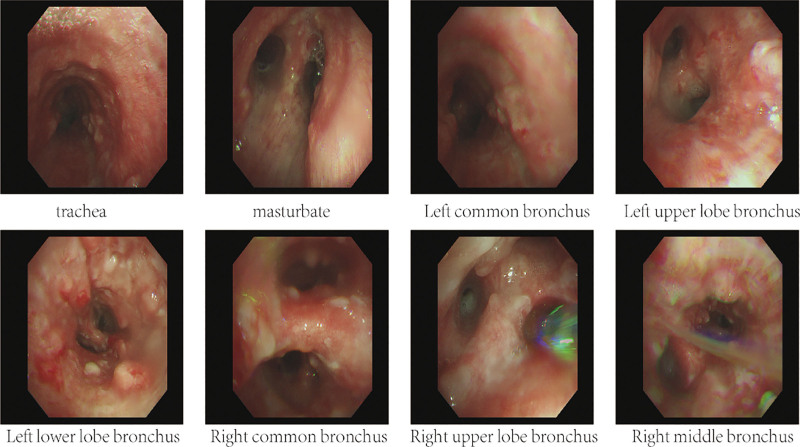
Sheath-like smoothness defines the mucosa of tracheal segments I and II. Nodular protrusions with noticeable calcification that extend into the lumen have been observed in the carina and tracheal segment III. The left main bronchus, left upper lobe, and left lower lobe all have diffuse nodular protrusions that extend into the lumen. The lumen of the left upper and lower lobes is blocked by extensive sputum plugs.

**Figure 3.**
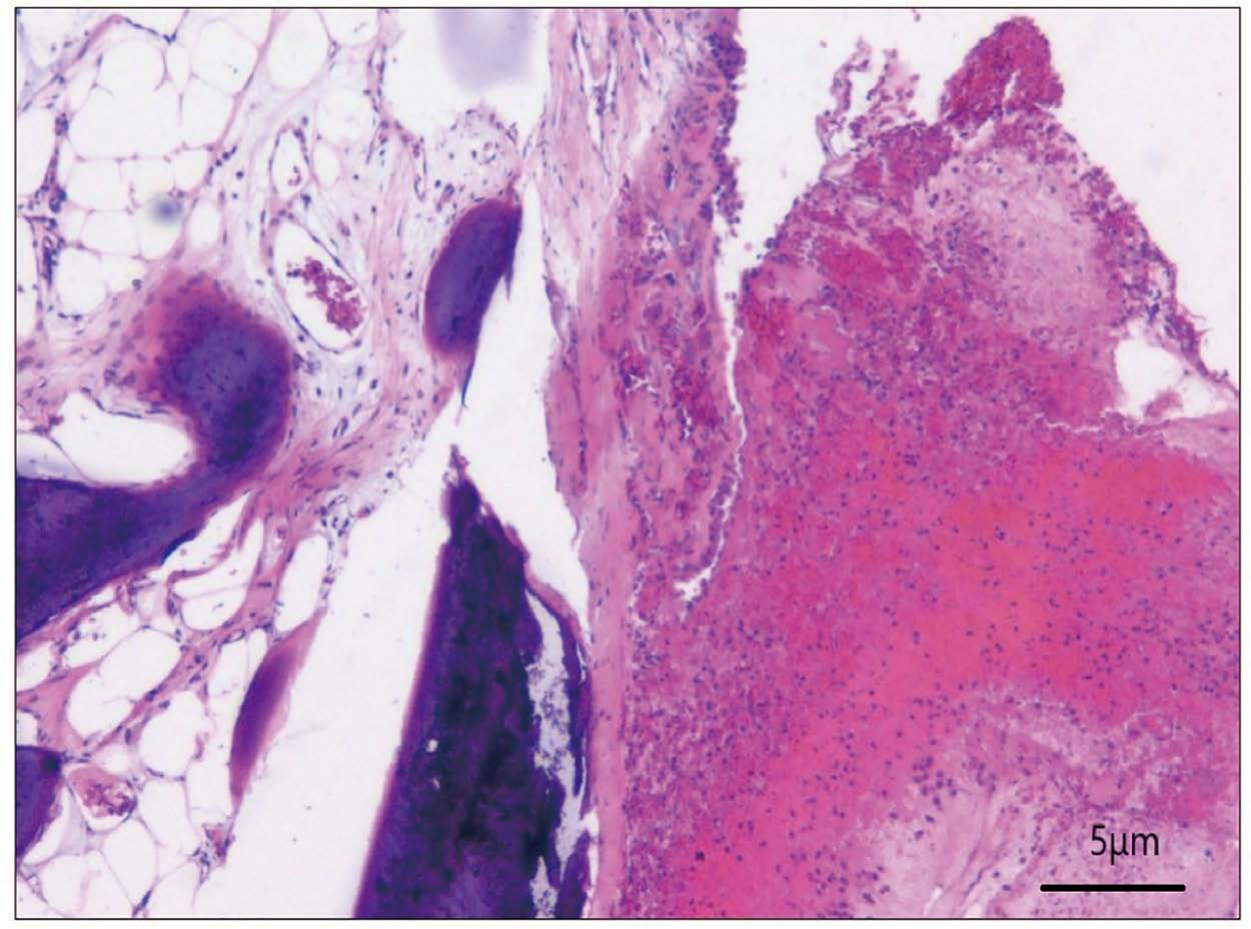
Shows HE stained tissue covered with complex epithelium, with some epithelium showing squamous metaplasia. Additionally, hyperplastic bone and fatty medullary tissue can be seen in the subepithelial mesenchyme. The image is magnified at 200 times. HE = hematoxylin-eosin.

A 55-year-old male, Han Chinese, previously engaged in renovation work, has a history of chronic cough, sputum production, and asthma for the past 10 years. He is capable of self-care in daily life and has not received any treatment. Approximately 20 days ago, he presented to an external hospital with worsening cough, sputum, and chest tightness accompanied by fever, where he received anti-infective treatment. A thoracic CT scan revealed a small amount of right pleural effusion and right lung atelectasis. Sputum culture results indicated the presence of *Klebsiella pneumoniae* and *Staphylococcus aureus*. He was initially treated with a combination of Meropenem and fluconazole for 1 week before being downgraded to ceftazidime. However, the patient’s symptoms of cough, sputum, and chest tightness worsened. On December 21, 2023, a sputum culture identified maltophilic narrow food monococcal bacteria, prompting a change in antibiotics to Cefoperazone-sulbactam combined with fluconazole. Despite this treatment, symptom relief was minimal, and the patient experienced increased chest tightness, restricted activities, and an increase in yellow purulent sputum. After visiting our hospital, the results were as follows: 1 to 3-β-D glucan levels were <10 pg/mL, the T-cell spot test (T-spot) was negative, and multiple sputum tests for acid-fast bacilli returned negative results. Additionally, no abnormalities were observed in autoimmune system-related tests, such as those for rheumatism and vasculitis, and no irregularities were found in tumor markers. The chest CT examination revealed enhanced blurring of the lung texture, point-like blurred foci along the bronchial lines, multiple bronchial wall thickening with calcification, and partial atelectasis in the middle and lower lobes of the right lung, as well as in the lower lobe of the left lung. No significantly enlarged lymph nodes were detected in the mediastinum. The thorax appeared symmetrical, with no definite effusion in the pleural cavity; however, the right pleura was diffusely thickened (Fig. 1). Further bronchial examination revealed normal vocal motility and smooth luminal mucosa in trachea I and II, exhibiting cuticular changes. In trachea III, there were nodular protrusions and ronchi with significant calcification, extending into the lumen. The left main bronchus, along with the upper and lower lobes of the left lung, displayed diffuse nodular protrusions encroaching into the lumen, accompanied by large amounts of sputum plugs in both lobes obstructing the airway. Following saline lavage, 6 mL of fluid was recovered from the lower lobe of the left lung for pathogenetic testing. Nodules were also observed protruding into the lumen of the right main bronchus, the right upper lobe, and the right middle segment bronchus. Hard tissue was identified in the biopsies from the ronchi, as well as the left upper lobe, left lower lobe, right upper lobe, and right middle segment bronchus (Fig. 2). Bronchoscopic freezing and laser treatment were performed concurrently. The examination of alveolar lavage fluid using targeted Next-Generation Sequencing (tNGS) revealed the presence of *Stenotrophomonas maltophilia* (14,167), *Pseudomonas aeruginosa* (2142), *Escherichia coli* (125), *Staphylococcus aureus* (66), human rhinovirus (8651), human herpesvirus type 7 (6977), and human herpes simplex virus type 1 (5595) in the alveolar lavage fluid (Tables 1 and 2). Histopathological examination of the tracheal biopsy indicated that the examined tissue was covered with complex epithelium, with some areas exhibiting squamous metaplasia. Additionally, hyperplastic bone and fatty medullary tissue were observed in the subepithelial mesenchyme, which was deemed a possible case of osteoblastic bronchiectasis (Fig. 3). Based on tNGS results, targeted eradication of primary pathogens (*Pseudomonas maltophilia*, *Pseudomonas aeruginosa*, *Staphylococcus aureus*, herpesvirus). The patient received anti-infective treatment comprising meropenem in combination with linezolid, acyclovir dispersible tablets, and levofloxacin. An anti-inflammatory regimen of methylprednisolone at 40 mg/day was also implemented, along with treatment using a salmeterol/ticloson inhaler for inhalation, cough suppression, and sputum resolution. The patient was subsequently discharged with reduced sputum volume and alleviation of chest tightness (Table 3). Regular follow-up examinations were undertaken as medically advised throughout the recovery period, with no signs of relapse observed within the 6-month interval.

**Table 1:** tNGS genetic test report of bacterial pathogens.

categorization	Bacteria genus	Bacterial species	Sequence number
Viruses	*Stenotrophomonas spp.*	*Stenotrophomonas maltophilia*	14,167
Viruses	*Pseudomonas*	*Pseudomonas aeruginosa*	2142
Viruses	*Escherichia*	*Escherichia coli*	125
Viruses	*Staphylococcus*	*Staphylococcus aureus*	66

tNGS = targeted next-generation sequencing.

**Table 2:** tNGS genetic test report of viral pathogens.

categorization	Bacteria genus	Bacterial species	Sequence number
Viral	Small RNA viruses	*Human Rhinovirus*	8651
Viral	*Herpesviridae*	*Human herpesvirus 7*	6977
Viral	*Herpesviridae*	*Human alphaherpesvirus 1*	5595

tNGS = targeted next-generation sequencing.

**Table 3:** Disease progression and treatment timeline.

Date	Examination/treatment	Outcome/symptoms
December 4, 2023	Chest CT scan, sputum culture	Minimal right-sided pleural effusion with partial atelectasis of the right lung. Isolation of Klebsiella pneumoniae and Staphylococcus aureus
December 8, 2023	Meropenem and fluconazole	Symptoms worsened, presenting with cough, expectoration and chest tightness
December 15, 2023	Ceftazidime therapy	Further deterioration with activity limitation and increased yellow purulent sputum
December 21, 2023	Repeat sputum culture testing	Isolation of Pseudomonas aeruginosa. Treatment adjusted to Cefoperazone-Sulbactam and Fluconazole
Following admission (dates detailed in multiple examinations)	Series of examinations and treatments	All tests negative or unremarkable. Chest CT reveals further lesions. Bronchoscopy shows nodules and sputum plugs. Pathology indicates osteoid proliferation
January 8, 2024	Meropenem, linezolid, acyclovir dispersible tablets, methylprednisolone, etc	Condition improved on discharge: reduced sputum volume, relieved chest tightness. Prescribed salmeterol/fluticasone inhaler

CT = computed tomography.

## 3. Discussion

Tracheobronchial osteochondrosis (TO) represents another rare airway disorder of unknown etiology.^[4]^ It is hypothesized to originate from fibrosis of submucosal elastic fibers, subsequently forming elastic cartilage followed by calcification and ossification. Alternatively, TO may result from the eventual ossification of ectopic osteochondromas arising from hyperplasia of tracheal cartilage rings.^[5]^ TO most commonly affects male patients aged 60 to 80 years. Clinical manifestations lack specificity, with typical symptoms including chronic cough, expectoration, hemoptysis, exertional dyspnea, wheezing, and recurrent pulmonary infections.^[6,7]^ These symptoms arise from airway irregularities caused by the pathology, increased airflow sensitivity, activation of the cough reflex, and accumulation of sputum due to the loss of normal ciliated columnar epithelium. The table below summarizes previously published cases associated with TO (Table 4).^[8–13]^

**Table 4:** Tracheobronchopathia osteochondroplastica: summary of published cases.

Reference	Age	Sex	Symptoms	Management	Key outcomes
PatelPM et al (2022)	41	Female	Progressive exertional dyspnea	Nd: YAG laser therapy was administered, followed by mechanical debulking.	Symptom relief; stable at 2 yr
SuatYeeLim et al (2021)	53	Male	Prolonged cough and recurrent, respiratory tract infection	Intravenous antibiotic therapy course	Partial improvement; stable at 1 yr
Yong Feng et al (2024)	51	Male	Malignant tumor of the lung, chronic expectoration of yellow and white sputum in substantial quantities	Right upper lobectomy	Symptom relief; stable at 4 mo
Giovanni Francesco Dall’Amico et al (2023)	50	Female	Asymptomatic (discovered during surgical anesthesia intubation)	No intervention was undertaken.	No intervention; stable monitoring
Jing Lai et al (2024)	63	Male	Persistent coughing with phlegm, accompanied by streaks of blood in the sputum	Anti-infective, hemostatic, coagulant, antitussive and expectorant	Partial improvement; stable at 2 yr
Maneesh Gaddamet al (2025)	64	Female	Asymptomatic (abnormal imaging findings)	Bronchoscopic biopsy	No complications

The diagnosis of TO relies heavily on imaging techniques such as CT scans, bronchoscopy, and histopathological examination. Chest CT scans typically reveal extensive calcification in the trachea and bronchi, along with calcified nodules that protrude into the airway. These nodules are usually found on the anterior and lateral walls of the airways, rarely affecting the vocal folds or tissues above them. In severe cases, there may be diffuse thickening of the tracheal walls, leading to fusion of the nodules and narrowing of the airway lumen. The gold standard for diagnosis is bronchoscopy.^[14]^ Tracheoscopy revealed the presence of numerous cartilaginous and/or bony nodules in the submucosa of the trachea, which protruded into the lumen. The nodules, which show cobblestone and stalactite cave-like features, are mainly located in the anterior and lateral walls of the trachea. Because the clinical presentation of the disease is nonspecific, submucosal biopsy is required to diagnose and differentiate it from other respiratory diseases, such as bronchopulmonary amyloidosis, atrophic chondromalacia, bronchial papillomatosis, bronchial calcification, and tuberculosis and sarcoidosis (e.g., nodular disease).^[15,16]^

Our patient had a history of renovation work and presented with recurrent symptoms including cough, sputum production, and chest tightness. The initial onset of symptoms was atypical. The external hospital diagnosed an acute exacerbation of chronic obstructive pulmonary disease and administered anti-infective and bronchodilator treatments; however, the patient’s symptoms did not significantly improve. Consequently, we conducted a further investigation into the underlying cause of the disease. A repeat chest CT scan revealed tracheal stenosis, indistinct foci along the bronchial alignment, and multiple bronchial wall thickening with calcification. Therefore, enhancing clinicians’ awareness of this condition is paramount. When respiratory symptoms such as coughing, wheezing, or hemoptysis persist despite routine clinical management, prompt bronchoscopy and pathogen testing should be performed. At the very least, bronchoscopy should be considered to rule out the possibility of Toxoplasma infection.

The current treatment for TO is mainly symptomatic, focusing on addressing symptoms such as infection, cough, airway secretions, and bronchospasm. This may include anti-infection medications, cough suppressants, airway secretion drainage, inhaled bronchodilators, and glucocorticoids. Studies have reported^[17]^ that inhaled glucocorticoids have the ability to reduce airway inflammatory response and reverse the chemotaxis of mucosal epithelial cells, potentially leading to therapeutic benefits. Cryotherapy and laser therapy can be applied directly to the affected area, precisely targeting diseased tissue to effectively minimize damage to surrounding healthy tissue. Cryotherapy induces cell death in the frozen lesion, thereby reducing the risk of bleeding; laser therapy, meanwhile, achieves haemostasis by coagulating blood vessels through high temperatures. Additionally, for respiratory distress caused by airway narrowing, bronchoscopic interventional therapy may be employed to improve ventilation function, alleviate symptoms, and enhance the patient’s quality of life.

## 4. Conclusion

TO is a rare benign disease with variability in clinical presentation and prognosis. It relies on bronchoscopy for diagnosis and treatment, which is supportive and symptomatic. The key to treating chronic inflammatory conditions is early recognition, which is critical to preventing disease complications and progression.

## Author contributions

**Data curation:** Xiaona Yin, Xi Wang, Xiaoyue Liu, Lijuan Zhou, Fangbin Du, Yinling Jiang, Dongmei Su.

**Formal analysis:** Xiaona Yin, Xi Wang, Xiaoyue Liu, Lijuan Zhou, Fangbin Du, Yinling Jiang, Dongmei Su.

**Writing – original draft:** Chuchu Xu.

**Writing – review & editing:** Xiaoqiong Wang, Yongsheng Wang.
